# Acute Mania

**Published:** 1908-06-27

**Authors:** 


### MENTAL DISEASES.

### ACUTE MANIA.

SYMPTOMS of acute mania (delirium) often appear very suddenly in cases that have been showing mild mental symptoms. The patient rapidly becomes excited, restless, noisy, and unmanageable. The first symptoms appear most frequently in the evening, and throughout the attack the patient is usually quieter in the morning, becoming more excited towards night. Loss of self-control and power of attention, carelessness of personal cleanliness, irrationality in conversation and behaviour, incoherence of ideas, sleeplessness, and irresponsibility are the most marked features. Delusions of various kinds are generally present, those of identity being common, but as a rule they change rapidly. On account of delusions the patient often takes a dislike to certain persons, usually those for whom, in the normal mental condition, most affection is felt. Hallucinations may be vivid and lead to impulsive action and violence. Very marked loss of emotional control appears at times, laughter or weeping being induced at will by the tone of voice in which the patient is addressed. In severe cases the patient becomes noisy, abusive, obscene, destructive, violent, and dirty. Certain physical signs usually accompany these mental symptoms. The pulse is small and rapid, and the blood pressure diminished; the temperature is normal or subnormal. The bowels are costive, the appetite is variable, and may be excessive, while loss of body weight always occurs. In females catamenia cease or become irregular.

There is another form of acute mania which is rare, but which deserves special mention, called acute delirious mania. In this form there is an exaggeration of mental symptoms, together with well-marked physical ones. Incoherence and insomnia are great, restlessness and resistiveness excessive, and the patient displays a total lack of appreciation of his surroundings and of anything that is done for him. Nervous symptoms—*e.g.* muscular tremor and subsultus—are prominent, the tongue is dry, sordes accumulate, food is persistently refused, there is rapid wasting, and (an important point) the temperature is raised. This condition resembles a typhoid state, with active maniacal symptoms.

The prognosis in cases of simple acute mania is good, though as to the length of the attack there is never any indication. The presence of physical disease or of epileptic or paralytic symptoms adds seriousness to the outlook. The prognosis in acute delirious mania is always extremely bad; even under early active and skilful treatment a fatal termination very frequently occurs.

In the treatment of acute mania special care must be taken that no impulsive act of the patient can lead to injury. Windows must be guarded, and anything with which he can damage himself or others must be removed. A trained mental nurse will manage the patient better, more easily, and more humanely than one who has not had special training in mental cases. A purgative should be given at once, unless specially contra-indicated. If he will stop in bed the patient should be kept there; but if he will not remain there, efforts to force him to do so will probably result only in active resistance and increased excitement. Mechanical restraint should not be employed, since it results in great struggling and a dangerous degree of fatigue, without benefit to the mental symptoms. A hot bath very often has a great sedative effect, and induces sleep. It is usually enjoyed by the patient, and, if the desired effect is obtained from it, should be used in preference to sedative drugs. He may be left in the bath for a long time if the temperature of the water be maintained, but under no circumstances should he be left alone whilst in it. Sedative drugs should always be avoided if possible, but, if necessary, sulphonal, trional, veronal, paraldehyde, bromides, chloral hydrate, cannabis indica, and hyoscyamus are the most useful. Morphia should not be given in mania: it only leads to constipation and a tendency to increased excitement. Hyoscine and hyoscyamine are to be avoided. Twenty grains of sulphonal or trional are usually satisfactory, whilst, if a hypnotic is required without sedative after effects, five grains of veronal or two drams of paraldehyde will be suitable. If it can be avoided drugs should never be administered in an insane patient's food, as this procedure often causes refusal of food, and may even give rise to a delusion that attempts are being made to poison him. If the appetite is good, plenty of easily digestible food should be given, and if there is a tendency to bolt it, special precautions must be taken. If sufficient food is not taken, recourse must be had to forcible feeding. In acute delirious mania early and frequent forcible feeding is necessary.

During convalescence a reactionary depression almost invariably occurs. The patient rapidly regains weight and strength, becomes less excitable, and finally appears normal. It is then that depression occurs, sometimes showing itself with startling suddenness by a determined attempt at suicide; and, unless a careful watch is kept, this attempt may be successful. This attack of mental depression may be of long or short duration, but it usually passes off and is followed by complete recovery.

